# MicroRNA-337 inhibits colorectal cancer progression by directly targeting KRAS and suppressing the AKT and ERK pathways

**DOI:** 10.3892/or.2022.8394

**Published:** 2022-08-25

**Authors:** Xuerong Liu, Yan Wang, Jinglong Zhao

Oncol Rep 38: 3187–3196, 2017; DOI: 10.3892/or.2017.5997

Following the publication of this paper, it was drawn to the Editors' attention by a concerned reader that various panels showing data from flow cytometric experiments in Figs. 2D and 5D were strikingly similar to data appearing in different form in other articles by different authors. Owing to the fact that the contentious data in the above article were already under consideration for publication, or had already been published, elsewhere when it was submitted to *Oncology Reports*, the Editor has decided that this paper should be retracted from the Journal. The authors were asked for an explanation to account for these concerns, but the Editorial Office did not receive a reply. The Editor apologizes to the readership for any inconvenience caused.

